# Peripheral neural interfaces: Skeletal muscles are hyper-reinnervated according to the axonal capacity of the surgically rewired nerves

**DOI:** 10.1126/sciadv.adj3872

**Published:** 2024-02-28

**Authors:** Vlad Tereshenko, Dominik C. Dotzauer, Martin Schmoll, Leopold Harnoncourt, Genova Carrero Rojas, Lisa Gfrerer, Kyle R. Eberlin, William G. Austen, Roland Blumer, Dario Farina, Oskar C. Aszmann

**Affiliations:** ^1^ Division of Plastic and Reconstructive Surgery, Massachusetts General Hospital, Harvard Medical School, Boston, MA, USA.; ^2^ Clinical Laboratory for Bionic Extremity Reconstruction, Medical University of Vienna, Vienna, Austria.; ^3^ Center for Medical Physics and Biomedical Engineering, Medical University of Vienna, Vienna, Austria.; ^4^ Center for Anatomy and Cell Biology, Medical University of Vienna, Vienna, Austria.; ^5^ Division of Plastic and Reconstructive Surgery, Weill Cornell Medicine, New York, NY, USA.; ^6^ Department of Bioengineering, Imperial College London, South Kensington Campus London, SW7 2AZ London, UK.; ^7^ Department of Plastic, Reconstructive and Aesthetic Surgery, Medical University of Vienna, Vienna, Austria.

## Abstract

Advances in robotics have outpaced the capabilities of man-machine interfaces to decipher and transfer neural information to and from prosthetic devices. We emulated clinical scenarios where high- (facial) or low-neural capacity (ulnar) donor nerves were surgically rewired to the sternomastoid muscle, which is controlled by a very small number of motor axons. Using retrograde tracing and electrophysiological assessments, we observed a nearly 15-fold functional hyper-reinnervation of the muscle after high-capacity nerve transfer, demonstrating its capability of generating a multifold of neuromuscular junctions. Moreover, the surgically redirected axons influenced the muscle’s physiological characteristics, by altering the expression of myosin heavy-chain types in alignment with the donor nerve. These findings highlight the remarkable capacity of skeletal muscles to act as biological amplifiers of neural information from the spinal cord for governing bionic prostheses, with the potential of expressing high-dimensional neural function for high-information transfer interfaces.

## Teaser

Skeletal muscles can amplify neural output from the spinal cord, enhancing high-information transfer for bionic prostheses.

## INTRODUCTION

Devastating injuries to the upper extremities pose a substantial challenge for the global healthcare system and require a comprehensive, interdisciplinary approach for counteracting their effects (*1*, *2*). In recent decades, bionic reconstruction has considerably advanced, becoming a viable therapeutic modality for restoring extremity function in cases where biological hand replantation or transplantation is not feasible (*3*–*6*). Recent surgical techniques, such as targeted muscle reinnervation (TMR), regenerative peripheral nerve interface (RPNI), and agonist-antagonist myoneural interface, have improved prosthetic control in upper and lower extremity by advancing the communication link between the nervous system and prostheses (*7*–*9*). Ideally, an ultimate biological interface should be able to incorporate and transfer neural information in a high-resolution manner to a prosthesis. However, while surgical techniques have emerged, technological advancement in robotics have greatly outpaced the capability of the biological interfaces to exploit the sophisticated highly tuned movements of a robotic limb (*5*). This mismatch results in an impaired capability of the patient to communicate with the machine and, thus, remains one of the most challenging issues in bionic reconstruction.

**Fig. 1.**
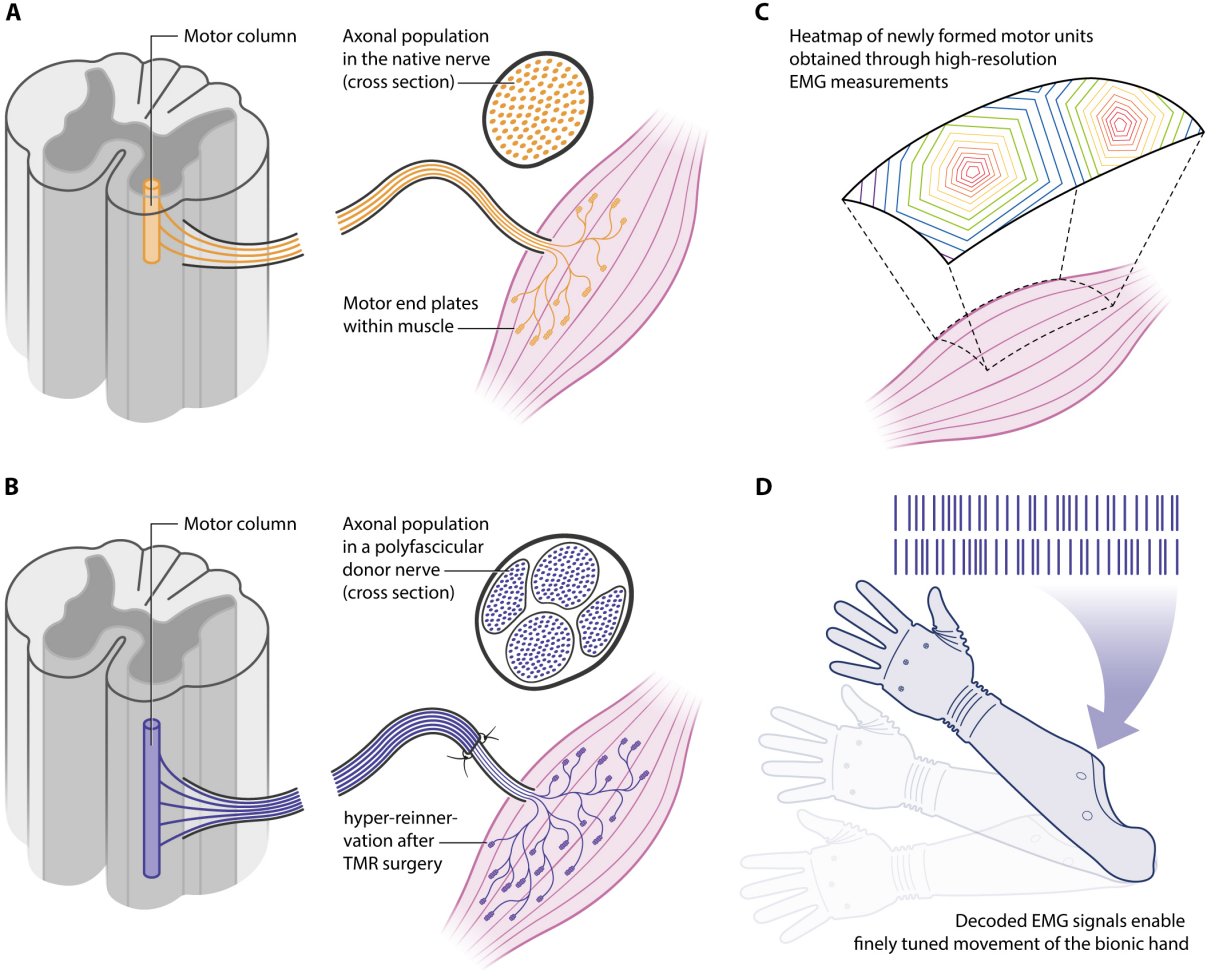
The skeletal muscle represents a biological interface for augmentation of neural signals. (**A**) The original neural input controlling a skeletal muscle can be surgically replaced by different neuronal sources. (**B**) The volume of surgically rewired neural input (i.e., the number of axons in the transferred nerve) can exceed the original neural capacity of the muscle (i.e., the number of axons in the nerve physiologically innervating the target muscle) by a significant factor. (**C**) This enables the reinnervated muscle to incorporate a much greater number of neural signals that originate from the spinal cord. (**D**) These signals can be decoded by source separation of the electric fields detected on the muscle [electromyography (EMG)], allowing for incorporating more neural commands and, thus, more intuitive control of a bionic prosthesis.

More than 22,000 motor neurons are responsible for dexterous movements of the human arm (*10*). Bionic reconstruction aims to emulate the natural dexterous movements by transferring this abundant neural information to the prosthesis. In patients suffering from traumatic amputations, malignancies, or brachial or lumbosacral plexus injuries, the neural pathways are either disrupted or partially missing in the injured extremity (*11*). Neural information, however, remains preserved within the remnants of non-impaired peripheral nerves of the amputation stump. TMR and RPNI amplify this neural input by redirecting it into residual or transplanted muscle tissue and subsequently retrieving the neural motor signals from the reinnervated muscles. The main aim of creating a muscle-based biological interface is to decipher as much information as possible from the activity of the motor neuron pools. For this purpose, it is preferrable to create a muscle interface with a high ratio of neural input (i.e., number of motor axons) to muscle volume (*5*). This is because modern methods for decoding muscle signals allow for the separation of the activity of individual motor neurons (Fig. 1) (*12*). However, despite the high plasticity properties of the muscle tissue, the relation between the number of nerve fibers transferred to a target muscle and the number of reinnervated fibers remains unclear.

Myoneural interfaces are a reliable bioscreen for neural information (*13*). Surgically redirected motor neuronal sources from another nerve can functionally reinnervate the target muscle, indicating its remarkable capacity for neuroplasticity. In this study, we analyzed the reinnervation capacity and changes in the physiological properties of a single muscle, originally controlled by a low number of motor neurons, using high- or low-motor neuron capacity donor nerves. The target muscle (sternomastoid) and transferred nerves (facial and ulnar) are not used for clinical nerve transfers in prosthetic fitting but, nonetheless, have been chosen in this study to reach a clear differentiation in axon numbers among the transferred nerves. This choice was made to identify an association between axonal count in the transferred nerves and reinnervated axons. The high-capacity nerve transfer demonstrated an almost 15-fold motor hyper-reinnervation compared to the low-capacity nerve transfer. Moreover, different neuronal sources redefined the molecular profile of the muscle fiber types, indicating changes in the muscle physiology. These results provide an association between the neural capacity of the donor nerve and the number of reinnervated motor axons, which is essential for the design of novel man-machine interfaces based on nerve transfers and myoelectric signal processing.

## RESULTS

### Functional reinnervation

**Fig. 2.**
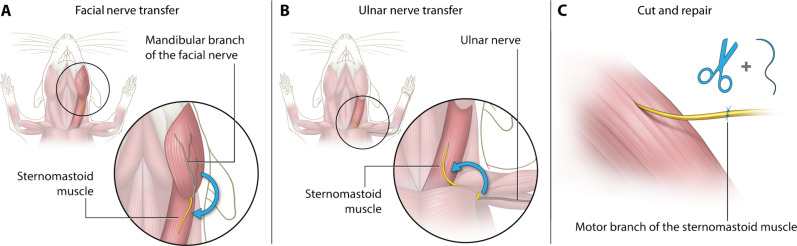
Experimental design for surgical neural input rewiring using donor nerves with high-, low-, or original axonal capacity. The sternomastoid muscle was used as a target muscle due to its large volume and native low neural input. Three groups were investigated: high-capacity nerve reinnervation (**A**), low-capacity nerve reinnervation (**B**), and reinnervation with the native neural input (**C**). (A) The mandibular branch of the facial nerve was used as a high-capacity donor nerve. The mandibular branch was transferred to the motor entry point of the sternomastoid muscle and coaptated using 11-0 suture. (B) The ulnar nerve was used as a low-capacity donor of neural input to the same target muscle. (C) The motor branch of the sternomastoid muscle was transected and immediately repaired using 11-0 suture to allow the reinnervation of the muscle via the original motor neural sources.

The sternomastoid muscle showed successful reinnervation regardless of the neural capacity or quality of the donor nerve. In the three groups of rats, (A) mandibular branch of the facial or (B) ulnar nerve transfers, as well as (C) cut-and-repair (cut&repair) procedures, were performed (Fig. 2). All animals (*n* = 69) survived the surgery and the 12-week follow-up period without any adverse effects. No deficits were observed in daily activities and no signs of severe pain, wound dehiscence, auto-mutilation, or infection were documented. The mean surgery time was 78 ± 12 min for the mandibular branch nerve transfer (MBNT) procedures, 72 ± 10 min for the ulnar nerve transfer (UNT), and 31 ± 9 min for the cut&repair surgery. The mean donor nerve length was 19.5 ± 1.6 mm for the MBNT and 35.6 ± 1.5 mm for the UNT procedures.

**Fig. 3.**
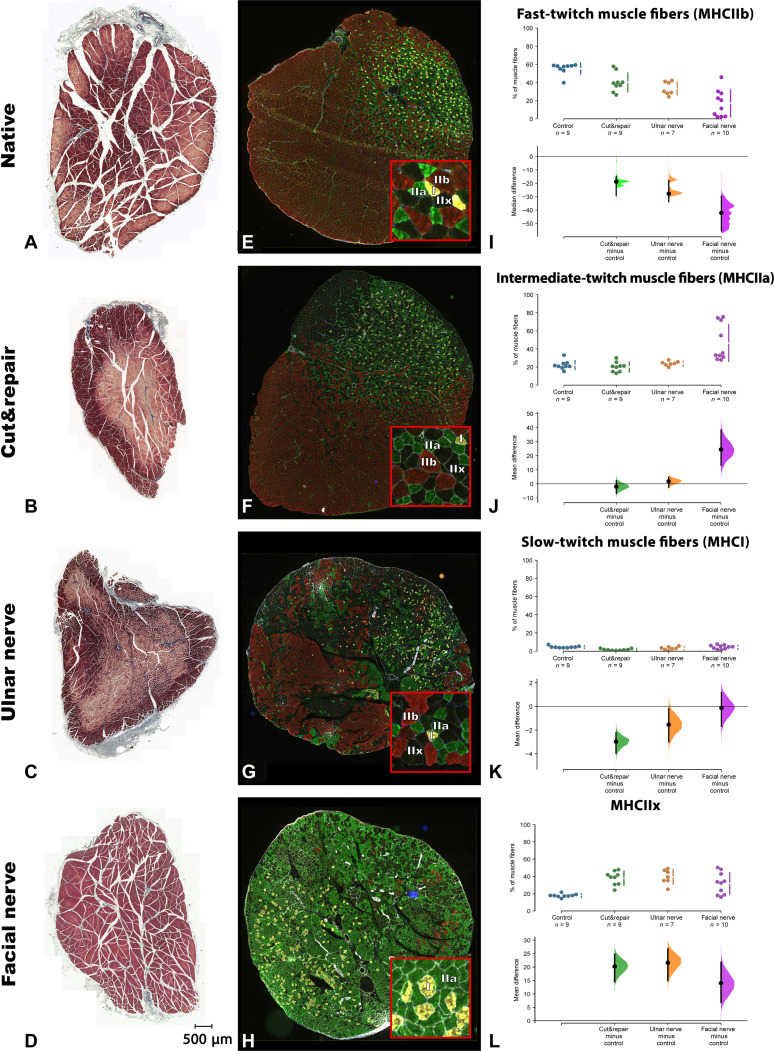
Morphological and muscle fiber–type changes after low- and high-capacity nerve transfer. The Masson-Goldner trichrome (MGT) staining was performed to evaluate the muscle atrophy on the cross sections of the sternomastoid muscle (*n* = 5 in each group) (**A** to **D**). No atrophy signs (angulated muscle fibers and scattered small fibers) were observed in the sternomastoid muscle after the cut&repair procedure (B), UNT (C), or facial nerve transfer (D) compared to the healthy muscle (A). The muscle fiber types were analyzed by immunofluorescence staining using antibodies against myosin heavy-chain I (MHCI), MHCIIa, MHCIIb, and MHCIIx and images acquired using confocal microscopy (**E** to **H**). The muscle fiber switch is depicted as a mean difference (or median difference for MHCIIb) for three groups (cut& repair, ulnar, and facial nerve transfers) against the healthy muscle on the Cumming estimation plots (**I** to **L**). The healthy sternomastoid muscle (*n* = 9) [(E) and (I)] consisted predominantly of fast twitch (55.4 ± 6.2%, red, MHCIIb) muscle fibers. Following the cut&repair procedure (*n* = 9), the ratio of intermediate-twitch fibers (MHCIIa; green) did not change significantly with a mean difference of −2.02 [95.0% confidence interval (CI), −6.7, 2.2] [two-sided permutation *t* test, *P* = 0.424 (J)], while the mean difference in MHCI ratio [−2.98 (95.0% CI, -−3.97, −2.21), yellow] and MHCIIb ratio [−18.8 (95.0% CI, −29.0, −14.8), red] fibers reduced [two-sided permutation *t* test, *P* < 0.01 (MHCIIb); two-sided permutation *t* test, *P* < 0.01 (MHCI) (F and K)]. Reinnervation using the high-capacity donor nerve (*n* = 10) changed the muscle fiber pattern compared to the control group by a significant reduction of fast-twitch [median difference is −42.1 (95.0% CI, −55.5, −29.7); two-sided permutation *t* test, *P* = 0.0048), increase in intermediate-twitch [mean difference is 24.4 (95.0% CI, 13.2, 38.4); two-sided permutation *t* test, *P* = 0.0002], and no significant decrease of slow-twitch muscle fibers [mean difference between control and facial nerve is −0.128 (95.0% CI, −1.69, 1.18); two-sided permutation *t* test, *P* = 0.878) [(H), (I), and (K)].

Gross morphological muscle reinnervation was confirmed using Masson-Goldner trichrome (MGT) staining (Fig. 3, A to D). No signs of atrophy (no scattered, small, and angulated muscle fibers; and no intramuscular fibrous tissue) were observed, and the muscle fibers appeared intact in all groups, indicating successful reinnervation. Functional reinnervation of the muscles was confirmed using electrical stimulation under different twitch frequencies (single twitch, 10, 20, 30, 40, and 50 Hz) (movies S1 and S2). Muscle contractions were observed in all groups (*n* = 9 per group), indicating functional reinnervation.

### Properties of the target muscle following reinnervation

Different motor neuron pools redefined the muscle properties by changing muscle fiber–type composition corresponding to the myosin heavy-chain (MHC) expression. The native sternomastoid muscle is composed of fast-twitch MHCIIb {58.1% [interquartile range (IQR), 54.1 to 58.5]}, intermediate-twitch MHCIIa [20.8% (IQR, 19.8 to 24.2)], slow-twitch MHCI [4.4% (IQR, 3.8 to 5.0)], and other hybrid or MHCIIx type [17.8% (IQR, 17.1 to 18.5)]. Following cut&repair surgery, the ratio of fast-twitch (MHCIIb) type increased, and the ratio of slow-twitch (MHCI) type decreased compared to the healthy muscle {unpaired median difference of −18.8 [95.0% confidence interval (CI), −29.0, −14.8] for MHCIIb and −2.98 (95.0% CI, −3.97, −2.21) for MHCI; two-sided permutation *t* test, *P* < 0.01} (Fig. 3). Following reinnervation with the low-capacity ulnar nerve, no significant difference in expression levels of MHC types was observed compared to the cut&repair model (two-sided permutation *t* test, *P* > 0.05) (fig. S1A). However, 12 weeks after reinnervation using the high-capacity cranial motor nerve, the sternomastoid muscle changed its muscle fiber–type profile significantly compared to the cut&repair model. In total, the sternomastoid muscle changed its expression profile toward a slower muscle by a decrease in fast-twitch muscle fibers [mean difference between is −23.0 (95.0% CI, −33.2, −11.4); two-sided permutation *t* test, *P* = 0.002] and an increase in intermediate-twitch [26.4 (95.0% CI, 15.3, 40.2); two-sided permutation *t* test, *P* = 0.0004] and slow-twitch muscle fibers [2.85 (95.0% CI 1.41, 4.15); two-sided permutation *t* test, *P* = 0.004] compared to the cut&repair model (fig. S1B).

### A multitude of motor neuronal sources can be surgically redirected to a low-capacity muscle

**Fig. 4.**
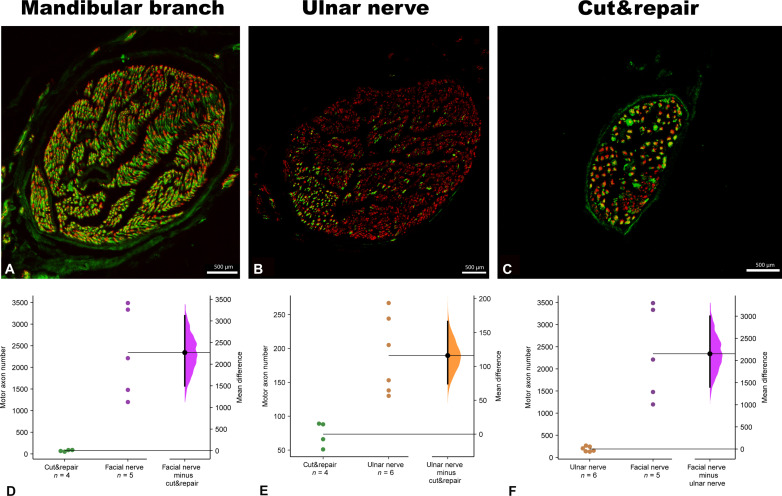
Quantitative analysis of redirected axons to the sternomastoid muscle. The cross sections of the transferred nerves (mandibular branch, ulnar nerve, and motor branch of the accessory nerve) 12 weeks following reinnervation (**A** to **C**). The mean difference in motor axonal power is shown in the Gardner-Altman estimation plots (**D** and **E**). The high-capacity facial nerve (*n* = 5) contained 30 times the motor axon number compared to the repaired original motor branch (*n* = 4) [mean difference of 2270 (95.0% CI, 1500, 3120); two-sided permutation *t* test, *P* = 0.0094 (D)]. The low-capacity donor nerve [ulnar nerve (*n* = 6)] contained a significantly higher motor axon number (190 ± 37) compared to the motor branch of the sternomastoid muscle (74 ± 9.4, *n* = 4) [mean difference of 116 (95.0% CI, 74.3, 116); two-sided permutation *t* test, *P* = 0.008 (E)]. Moreover, the facial nerve contained 12 times the motor axon number compared to the ulnar nerve [mean difference of 2150 (95.0% CI, 1400, 3000); two-sided permutation *t* test, *P* = 0.0094 (F)].

We observed that a single target muscle can incorporate a 15-fold amount of its original innervating axons after a high-capacity nerve transfer. Analysis of the original neural input to the sternomastoid muscle revealed 289 ± 29 of total axon number with 66 ± 9.4 (23%) somatic efferent nerve fibers in the motor branch of the accessory nerve. The high-capacity mandibular branch of the facial nerve contained 2248 ± 353 total axons of which 2173 ± 329 (96.7%) were motor axons, whereby the ulnar nerve contained 1728 ± 408 total axons, of which 153 ± 37 (8.9%) were motor axons. The initial discrepancy in the motor axon numbers between two nerves pinpoints the rationale for using these donor nerves to investigate high- and low-capacity reinnervation in this study (Fig. 4).

After reinnervation, the neural input was quantified on the axonal and neuronal levels. The donor nerves (motor branch of the accessory nerve and mandibular branch of the facial nerve and ulnar nerve) were analyzed after reinnervation. Twelve weeks after the reinnervation, the number of motor [choline acetyltransferase (ChAT)–positive] axons in the motor branch of the accessory nerve was 73.5 ± 18.4 of a total of 418 ± 154 axons. The total axon number was greater in the mandibular branch of the facial nerve (2697 ± 715) and in the ulnar nerve (3338 ± 532) after the surgery. Moreover, the number of cholinergic axons (motor) was significantly higher in the mandibular branch of the facial nerve compared to the motor branch of the accessory nerve [mean difference of 2270 (95.0% CI, 1500, 3120); two-sided permutation *t* test, *P* = 0.0094] and more than twofold higher in the ulnar nerve [mean difference of 116 (95.0% CI, 74.3, 116); two-sided permutation *t* test, *P* = 0.008] (Fig. 4D). This provided morphological evidence of a greater number of reinnervated fibers via the high-capacity donor nerve.

**Fig. 5.**
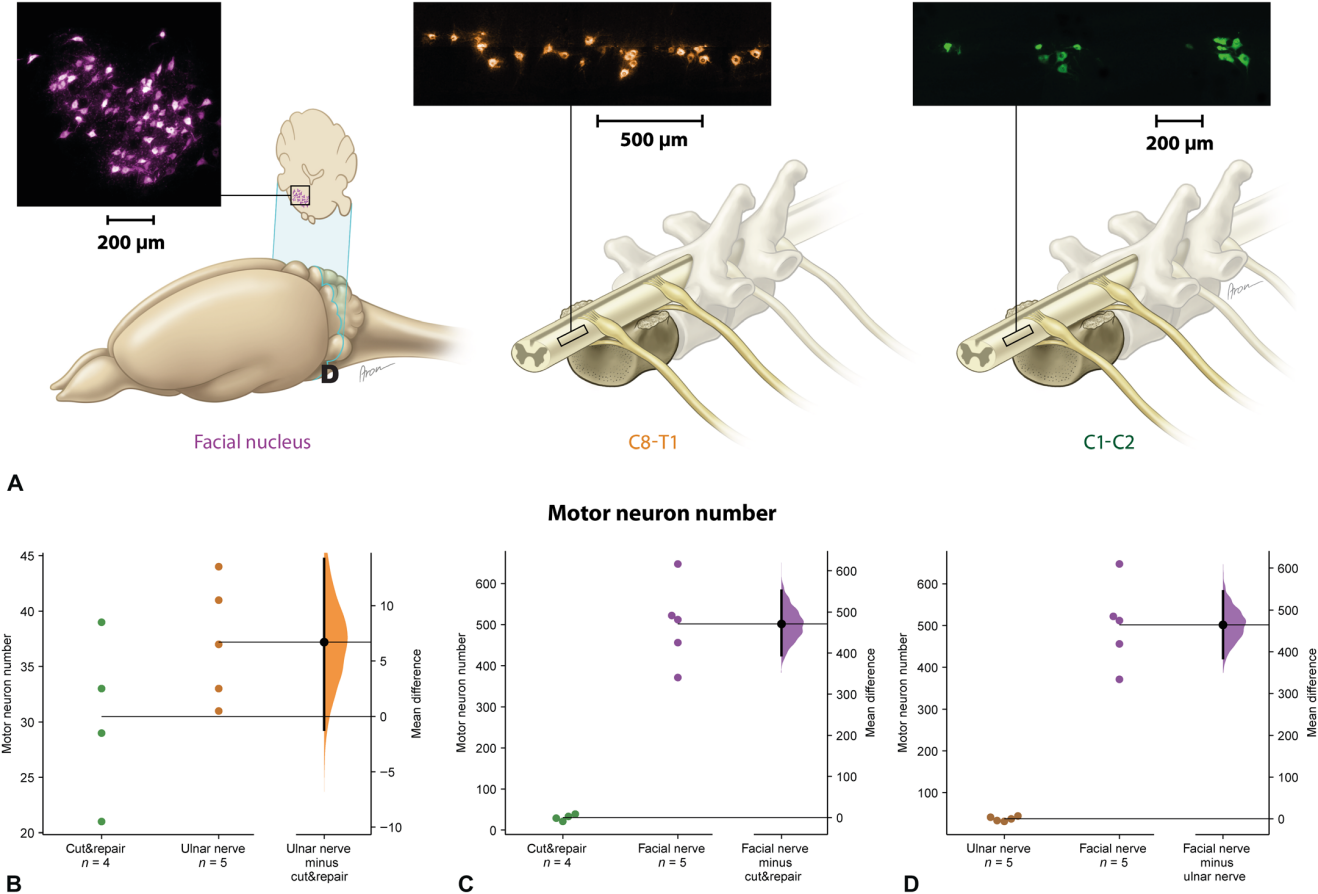
Analysis of surgically redirected neural sources at the CNS level. The motor neural sources following reinnervation were analyzed by retrograde tracing using intramuscular application of Fast Blue in all three groups (**A**). In the facial nerve transfer group, the abundance of motor neurons traveling via the facial nerve were traced back into the facial nucleus. In the UNT group, the spinal cord segment spanning from C8 to T1 was harvested, and the motoneurons responsible for reinnervating the sternomastoid muscle within the C8-T1 motor column were quantified. In the cut&repair group, the spinal cord segment spanning from C1 to C2 was harvested, and the motor neurons responsible for the native innervation of the sternomastoid muscle were quantified. The mean difference in viable motor neuronsis shown in the Gardner-Altman estimation plots (**B** to **D**). No significant difference was observed between the cut&repair and ulnar nerve groups [mean difference of 6.7 (95.0% CI, −1.2, 14.2); two-sided permutation *t* test, *P* = 0.137 (B)]. A significantly higher number of motor neuron axons traveling from the facial nerve was observed compared to the cut&repair procedure with a mean difference of 471 (95.0% CI, 394, 552) [two-sided permutation *t* test, *P* < 0.0001 (C)] and ulnar nerve with a mean difference of 464 (95.0% CI, 385, 545) [two-sided permutation *t* test, *P* < 0.0001 (D)].

To analyze neuronal sources that re-established the neuromuscular junctions of the sternomastoid muscle, retrograde axonal tracer (Fast Blue) was applied directly into the muscle 12 weeks after reinnervation. The number of motor neuronal sources reinnervating the sternomastoid muscle was more than a 10-fold amount of the neuronal sources after UNT [502 ± 101 versus 37.2 ± 5.4; mean difference of 464 (95.0% CI, 385, 545); two-sided permutation *t* test, *P* < 0.0001] and 15× after cut&repair surgery [502 ± 101 versus 30.5 ± 7.6; mean difference of 471 (95.0% CI, 394, 552); two-sided permutation *t* test, *P* < 0.0001] (Fig. 5). This highlighted the remarkable capacity of the sternomastoid muscle for receiving a high amount of neural information according to the donor nerve.

### Muscle as bioscreen of neural signals

**Fig. 6.**
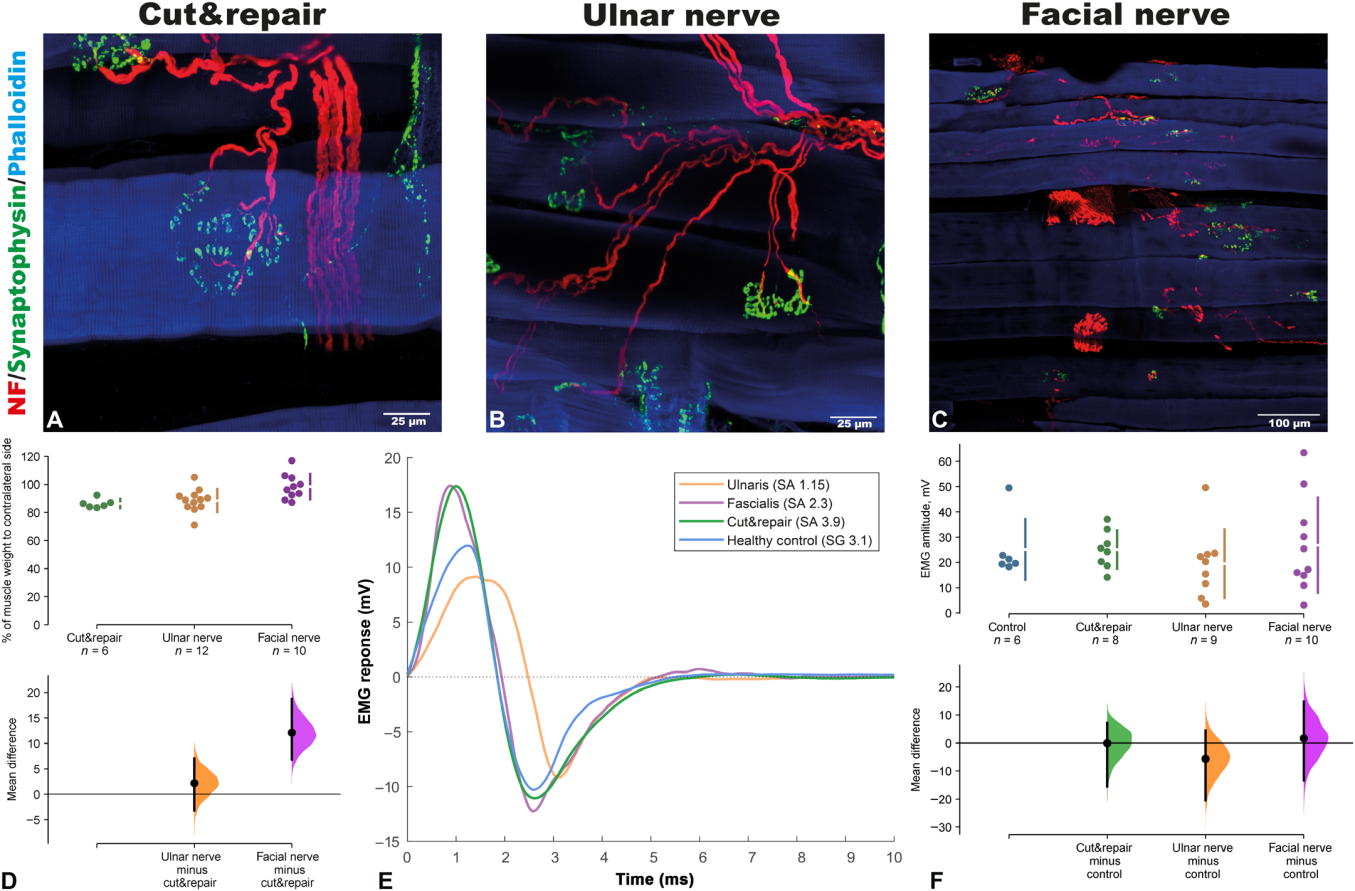
Neurobiological evidence for functional integration of high volume of neural input after high-capacity nerve transfer. Whole-mount staining of the reinnervated muscle after reinnervation using different donor nerves shows functional synaptic transmission within the de novo formed neuromuscular junctions [(**A**) cut&repair, (**B**) ulnar nerve, and (**C**) facial nerve (*n* = 4 in each group)]. Higher muscle weight following reinnervation in the facial nerve group compared to the cut&repair group shows correlation between the high number neural input and higher weight of the reinnervated muscle (two-sided permutation *t* test, *P* = 0.006) (**D**). Functional transmission of the neuromuscular junctions was confirmed using electromyographic recordings (**E**). The similar EMG amplitude in the facial nerve group compared to the control indicates a similar number of activated muscle fibers in the two cases, although the facial nerve group presented a higher number of de novo formed NMJs [mean difference of 1.65 (95.0% CI, −13.3, 14.8); two-sided permutation *t* test, *P* = 0.851) [(E) and (**F**)].

Surgically redirected neuronal sources established functional motor units that produced reliable myosignals (Fig. 6, A to C). The muscle weight ratio between the operated sternomastoideus and contralateral healthy muscles was significantly higher in the group after the high-capacity nerve transfer (MBNT) (98.4 ± 9.0%) compared to the cut&repair model (86.3 ± 3.2%) with the mean difference of 12.1% (95.0% CI 6.86, 18.7) (two-sided permutation *t* test, *P* = 0.006), while no difference was observed between the UNT (88.5 ± 8.2%) and cut&repair groups [2.2% (95.0% CI, −3.22, 7.01); two-sided permutation *t* test, *P* = 0.54; Fig. 6D]. Using a whole-mount immunofluorescent technique, the donor nerves were identified at the motor entry point arborizing into the muscle and reinnervating the muscle fibers (Fig. 6). The axonal endings showed synaptophysin colocalization with alpha-bungarotoxin, indicating active synaptic transmission of the neuromuscular junctions (Fig. 6, A to C). The density of the neuromuscular junction in the reinnervated muscle compared to the native suggested reorganization of the muscle into a large number of smaller motor units (Fig. 6C).

Activation of newly established motor units of the reinnervated muscle could be detected using invasive electromyography (EMG). No significant difference was observed in the amplitude of the compound potential of the activated motor units after MBNT (26.8 ± 18.8 mV) compared to the amplitude on the control contralateral side (25.1 ± 12.0 mV), with a mean difference of 1.65 mV (95.0% CI, −13.3, 14.8) (two-sided permutation *t* test, *P* = 0.851) (Fig. 6, E and F). The compound EMG amplitude depends on the number of innervated muscle fibers, not on the number of motor units. The similar amplitude of EMG in the facial nerve group with respect to control indicates the reinnervation of the majority of the muscle fibers and their organization in a different number of motor units, with a higher number of de novo formed neuromuscular junctions (NMJs) in the facial nerve group.

## DISCUSSION

In extremity amputations, the biological effectors (e.g., the hand) can be supported or replaced by modern bionic prosthesis (*5*). Despite the rapid rise in prosthetic technology allowing for even higher dexterity than the human hand, the greatest challenge remains converting motor commands into machine language. Hence, a mismatch emerges between biological signals and non-biological prosthetic effectors. The most notable finding of this study is that a skeletal muscle can be reinnervated by a number of axons multiple times its initial physiological innervation, following a high-capacity nerve transfer. This indicates the vast interfacing capabilities of a skeletal muscle by unfolding neural information from the spinal cord and providing it for the control of a prosthetic device.

The ability of a skeletal muscle for reinnervation regardless of the origin of the motor neurons is remarkable and demonstrates its high capacity for plasticity (*14*). Atrophy of the skeletal muscle can be prevented by timely reinnervation using an uninjured nerve that leads in most cases to complete functional recovery (*15*). Regenerating axonal endings of the donor nerve connect to the muscle fibers to form de novo neuromuscular junctions, leading to the reestablishment of motor units (Fig. 6) (*13*). Our findings indicate that a skeletal muscle can be reinnervated by a number of motor axons at least 10 times greater than its physiological innervation (Fig. 5). This motor neuron pool becomes responsible for muscle innervation and dictates the firing pattern of the newly established motor units, leading to specific spatiotemporal muscle contractions. The muscle contractile properties, on the other hand, depend on the muscle fiber types and their distribution within the muscle (*16*, *17*). The expression pattern of the MHC types is determined by the motor neurons innervating the skeletal muscle. We showed that the repair of the original motor branch itself caused a significant shift in the MHC-type configuration in the sternomastoid muscle (Fig. 3). This, however, may be explained by the reorganization process of the muscle fibers during the regeneration of the reconnected axons (*18*). A significant shift in the expression pattern of the MHC types after rewiring the motor neuron pool from the high-capacity nerve to the sternomastoid muscle emphasizes that an alien motor neuron pool (cranial versus spinal neural sources) can redetermine the expression pattern of MHC types within the reinnervated muscle. Hence, the redirected axonal population can supercharge the target muscle with an abundance of biological signals as well as orchestrate its contractile properties.

In most clinical scenarios with devastating injuries of the extremity, a prosthetic restoration is a suitable solution following amputation (*6*). As outlined above, one of the most challenging issues is the decoding of the neural signals in terms of movement instructions to the prosthetic device (*5*). Neuromuscular interfacing modalities can be used for this purpose. Because TMR or RPNI have become a common approach for segmentation and augmentation of the biological signals within the muscle tissue, it remains crucial to select the donor nerves conveying neural input responsible for specific movement patterns. However, these neural signals should find “a new home” within a skeletal muscle to be decoded and integrated into the machine language. Only a little is known about the muscular capacity for the integration of complex neural information from the peripheral nerves (*19*, *20*). In the most deleterious clinical scenarios, the remnant muscle tissue following amputation is scarce, providing little room for maneuvering in the amplification of neural input for neuromuscular interfacing. Our findings shed light on the unprecedented capacity of a skeletal muscle to integrate a multifold neural input compared to its original supply (Fig. 5). Selective nerve transfer is mostly performed using one single nerve for one single muscle to achieve selective functional muscle reinnervation (*7*). However, to provide more elaborate control of a prosthetic device, decoding the neural input from all three different nerves (radial, ulnar, and median) responsible for the whole range of movement of the hand would be preferrable. The first experimental model for multiple nerve transfer for augmentation of the biological signals has been recently proposed, indicating that two separate nerves are capable of functional reinnervation of a single muscle (*21*). Thus, signal amplification using TMR can be performed by transferring a high neural input load to a single target muscle creating a high-fidelity bio screen for neuromuscular interfaces. Our data provide a neurobiological basis for the translation of this approach to patients with amputations and limited muscle tissue available for neuromuscular prosthetic interfacing.

While we showed that a single skeletal muscle can integrate a high volume of neural information, deciphering patients’ complex movement intents remains a challenging issue. Our data showed that a high number of neuronal sources can reestablish a high density of neuromuscular junctions within the target muscle (Fig. 6C). Thus, an increased motor unit number leads to a higher density of reorganized functional units within the same muscle volume. Decoding the high-density neural information distributed within one single muscle requires advanced interfacing systems (*22*), source-separation algorithms (*23*, *24*), and spatial pattern-recognition systems encompassing distinctive movement instructions (*25*). Recent advances in pattern-recognition systems for surface EMG (*26*, *27*) along with promising outcomes from wireless intramuscular sensors (i.e., implantable myoelectric sensor, Myoplant, or myoelectric implantable recording array) may allow deciphering of complex motor commands from several neural sources (*28*–*32*).

In conclusion, our findings indicate that the skeletal muscles mirror neural information incoming from the spinal cord. Surgical rewiring of this abundant neural input into a single target muscle can unveil the complex network of neural data from the spinal cord and transform it into the myoneural code. These neural signals can be decoded and mapped in a high-resolution manner according to the motor commands using advances in bioengineering and machine learning to create a reliable biological interface for high-fidelity control of a bionic prosthesis.

## MATERIALS AND METHODS

### Animals

Sixty-nine Sprague-Dawley rats (males, aged 8 to 10 weeks, weighing ∼250 g) were used in this study. Rats were stratified into three groups: cut&repair surgery, facial nerve (MBNT), and ulnar nerve transfers. In each group, 12 weeks were allowed for muscle reinnervation. After the surgery, the electrophysiological tests were performed on the operated and contralateral sides, following muscle harvesting, muscle weight analysis, and muscle fiber typing (*n* = 9 in each group). Retrograde labeling along with nerve harvesting for quantification of neural input was performed in each group 12 weeks after the surgery (*n* = 5 in each group), and the control nerve samples were harvested on the contralateral side. Histological analysis of the muscle including MGT (*n* = 5 in each group) as well as whole-mount staining was performed (*n* = 4 in each group) 12 weeks after the surgery. Approval was obtained from the ethics committee of the Medical University of Vienna and the Austrian Ministry for Research and Science (reference number 2021-0.155.204).

### Nerve transfer models

Three different surgical models were established to investigate the capability of a skeletal muscle to incorporate variable neural load (Fig. 2). Here, the sternomastoid muscle was used as a target muscle due to its low original neural load and high muscle surface area. In the first model, the motor branch of the accessory nerve was transected and immediately coaptated at the motor entry point. In the high-capacity nerve transfer model, the mandibular branch of the facial nerve was used as a donor nerve due to its high axonal load. The mandibular branch was transected proximal to the insertion of the masseter muscle or proximal to the bifurcation point of the mandibular branch and redirected caudally toward the sternomastoid muscle, where it was coaptated in the same manner as in the cut&repair group. For the low-capacity nerve transfer, the ulnar nerve was used. The nerve was transected distal to the dorsal branch, muscle branches (flexor carpi ulnaris and flexor digitorum profundus) are cut, and the distal ulnar nerve is transposed proximally to the ipsilateral sternomastoid muscle in the avascular plane under in the pectoral major muscle. All nerves are coaptated in the same manner to the motor entry point of the denervated sternomastoid muscle using 11-0 (Ethilon, Ethicon, Johnson & Johnson Medical Care) sutures.

### Muscle analysis

#### Muscle fiber typing

Morphological changes at the muscular level after nerve transfer were analyzed by muscle weight measurement, MGT staining, and muscle fiber typing. Muscle weight was analyzed to assess the gross effects of reinnervation using a microscale. Right after the muscle wight assessment, the muscles were embedded in O.C.T. compound (Tissue-Tek, Sakura Finetek, CA, USA), frozen in liquid nitrogen–cooled isopentane, and stored at −80°C for a minimum of 24 hours. Embedded samples were cut into 10 μm, and muscle fiber–type staining was applied as described previously (*33*). Briefly, antibodies against different MHC types were applied: MHCI (BA-F8; 1:50), MHCIIa (SC-71; 1:600), and MHCIIb (BF-F3; 1:100) were used. Subsequently, the entire cross sections of the muscle were acquired using a whole-slide scanner (Vectra Polaris, Akoya Biosciences) and subsequently analyzed using the A.I. Halo imaging analysis platform version 3.2.1851.421 (Indica Labs) by one independent investigator (V.T.).

For the MGT staining, the muscles were harvested and immediately stored in 4% paraformaldehyde (PFA) tubes for up to an additional 24 hours before being rinsed with phosphate-buffered saline (PBS) for 24 hours. The samples were embedded in paraffin for MGT staining. For this purpose, samples were placed in a tissue cassette, dehydrated (35 min in 100% ethanol and 90 min in isopropanol and paraffin each) using a microwave tissue processor (KOS, Milestone, Italy), and then embedded in paraffin. Afterward, the samples were cut into 4-μm sections and bound to microscope slides. The sections were then subjected to MGT staining according to the protocol previously described (*34*). We evaluated muscle atrophy on the basis of the following atrophy signs: intramuscular fibrous tissue; small, scattered, and angulated muscle fibers; or nuclear bags, which appear as clumps of nuclei encircled by the remaining sarcolemmal membrane (*35*, *36*).

#### Whole-mount staining

PFA-fixed muscles were further processed for cryoembedding according to the guidelines of Blumer *et al.* (*37*). Briefly, following fixation, muscles were washed in PBS and then cryoprotected in graded concentration of sucrose (10, 25, and 40%) in PBS containing 0.05% sodium azide to avoid bacterial and fungal contamination. Then, the muscles were frozen and stored at −80°C. Frozen sections were cut at 200-μm thickness, parallel to the muscle surface. Before immunolabeling, sections were incubated with 10% normal goat serum. After that, sections were incubated with the primary antibodies against neurofilament (NF; AB5539, Merck Millipore) at a concentration of 1:2000 and antibodies against synaptophysin (MAB329, Merck Millipore) at a concentration of 1:500 for 48 hours at room temperature. Thereafter, sections were washed in PBS and incubated with the Alexa Fluor 488– and Alexa Fluor 568–conjugated secondary antibodies. Muscle fibers were counterstained with Alexa Fluor 647–conjugated phalloidin.

Fluorescently labeled sections were analyzed with a confocal laser scanning microscope (CLSM Olympus FV3000). Images were captured with dry lenses of ×10 and ×20 magnification and oil lenses of ×40 magnification. A series of virtual confocal laser scanning microscopy sections of 1-μm thickness were cut through the structures of interest. Each section was photo-documented with a 1024 × 1024 pixel resolution, and three-dimensional projections were rendered using ImageJ software (National Institutes of Health, Bethesda, MA, USA). Triple-colored images were generated using lasers with excitation wavelengths of 488, 568, and 640 nm.

### Nerve analysis

The neural components responsible for functional muscle reinnervation were first quantified at the nerve level. Antibodies against specific molecular markers were applied to distinguish between different axonal types. Nerve samples from the motor branch to the sternomastoid muscle, ulnar nerve, and mandibular branch on both sides were harvested 12 weeks after the surgery and immediately fixated by immersion in 4% PFA diluted in 0.1 M PBS (pH 7.4) at +4°C for 24 hours. Afterward, the samples were processed and cut into 10-μm-thick cross sections as described previously (*38*). The immunofluorescence staining protocol using antibodies against NF and ChAT was used to quantify motor and sensory axonal components reinnervating the target muscle. Briefly, chicken anti-NF (AB5539) and goat anti-ChAT (AB144P) were used as primary antibodies at a concentration of 1:2000 and 1:100, respectively. Secondary antibodies conjugated with Alexa Fluor 488 or 568 were obtained from Thermo Fisher Scientific (Waltham, MA, USA) and were used at a concentration of 1:500. Images of nerve cross sections were acquired using a fully integrated imaging system (TissueFAXS; TissueGnostics, Vienna, Austria). Automated quantification of axons within the nerve samples was performed using StrataQuest version 5.1.249 and TissueQuest version 4.0.1.0128 (TissueGnostics, Vienna, Austria) as described previously (*10*, *39*).

### Retrograde tracing of neural input

The neuronal input innervating the target muscle was determined by retrograde tracing from the muscle into the central nervous system (CNS). This approach allows for quantification of viable motor neurons contributing to reestablishing functional NMJs in the reinnervated muscle (*40*). The sternomastoid muscle was exposed 12 weeks following the nerve transfer surgery. Using a 10-ml Hamilton microsyringe (catalog no. 7635-01, Hamilton Bonaduz) with a small hub removable needle (30 gauge; catalog no. 7803-07, Hamilton Bonaduz), 10 ml of 2% Fast Blue (catalog no. 17740-5, Polysciences) was injected in both muscles. To prevent any leakage from the muscles, the needle was left within the muscle for 30 s followed by slow withdrawal. One week after the tracer application, left ventricular perfusion was performed as described above. Depending on the group, we harvested specific regions of the CNS (brain for facial nerve transfer, and spinal cord from C8 to T1 level for UNT and from C1 to C2 level spinal cord for the cut&repair group) (*41*, *42*). The spinal cord and the brain stem were harvested and stored in 0.1 M PBS for 48 hours. After dehydration in increasing sucrose/PBS solutions (10, 25, and 40%), the samples were cryofixed and cut using a cryostat (Leica, Germany) into 50 mm. The number of labeled neurons was quantified by one trained observer (V.T.) in each sample harvested CNS sample for each group. To avoid double counting of perikarya, the average nucleolus diameter was calculated to apply the Abercrombie correction as described previously (*33*).

### Electrophysiology

Measurements of electrophysiological activity were conducted in all three groups. To evaluate the functional characteristics of the reinnervated sternomastoid muscle, a custom-made pulse generator (MiniVStim 18B, Competence Team for Implanted Devices, Center of Medical Physics and Biomedical Engineering, Medical University of Vienna, Austria) was used to electrically stimulate the respective nerves. A custom-built bipolar stainless steel hook electrode, with a 1.5-mm distance between hooks, was used to deliver monophasic current pulses with passive exponential charge balancing to the nerve. The current pulses had a supramaximal amplitude of 2 mA and a phase width of 258 μs. Muscular contractions were elicited by short (500 ms) bursts of stimulation at different frequencies (single twitch, 10, 20, 30, 40, and 50 Hz). A rest period of 3 s was incorporated between consecutive contractions.

In addition to visually observing the muscular contractions, compound muscle action potentials (CMAPs) were recorded for each contraction using 29-gauge needle electrodes (model: MLA1213, AD-Instruments, Sydney, Australia). The active EMG electrodes were placed along the fiber direction and centered across the mid-length of the muscle belly, with a separation of ~7 mm between them. A ground electrode was placed percutaneously in a nearby inactive area. The CMAPs were measured using a biosignal amplifier (BioAmp FE231, AD-Instruments, Sydney, Australia) and recorded by a data acquisition system (Powerlab 16/35, AD-Instruments, Sydney, Australia). The peak-to-peak amplitudes of the first CMAP of each contraction were determined using MATLAB R2010a (MathWorks, Natick, MA, USA) and averaged for each measured muscle.

### Statistical analysis

Descriptive statistics was used for surgery time and donor nerve length. In all trials, normal distribution was checked with the Kolmogorov-Smirnov test. The parametric data were compared between control healthy muscles, cut&repair, ulnar nerve, and mandibular branch groups using an unpaired *t* test. Nonparametric data [i.e., percentage of fast (MHCIIb) muscle fibers] were compared between the groups using Mann-Whitney *U* test. Results with a *P* value of <0.05 were considered significant. Data were analyzed using GraphPad Prism 8.0.2 (GraphPad Software, San Diego, CA, USA). Estimations Statistics (www.estimationstats.com/#/) was used to assess effect size and mean or median differences between groups (*43*). We used Gardner-Altman estimation or Cumming estimation plots for visualizing individual values in each group and bootstrap resampling techniques to calculate 95% confidence intervals and mean or median differences. In each iteration, 5000 bootstrap samples were taken; the confidence interval was bias-corrected and accelerated (*43*). For each permutation *P* value, 5000 reshuffles of the control and test labels were performed, which represent the likelihood of observing the effect size, if the null hypothesis of zero difference is true.
