# Supplementary material for: Peripheral neural interfaces: Skeletal muscles are hyper-reinnervated according to the axonal capacity of the surgically rewired nerves
Source: Sci Adv. 2024 Feb 28;10(9):eadj3872. doi: 10.1126/sciadv.adj3872 (PMC10901366; doi:10.1126/sciadv.adj3872)
Supplement: Supplementary file 1 — Fig. S1 Legends for movies S1 and S2 [file sciadv.adj3872_sm.pdf]

Science Advances
AAAS

Supplementary Materials for

Peripheral neural interfaces: Skeletal muscles are hyper-reinnervated according to the axonal capacity of the surgically rewired nerves

Vlad Tereshenko *et al.*

Corresponding author: Vlad Tereshenko, vtereshenko@mgh.harvard.edu;
Oskar C. Aszmann, oskar.aszmann@meduniwien.ac.at

Sci. Adv. **10**, eadj3872 (2024)
DOI: 10.1126/sciadv.adj3872

The PDF file includes:

Fig. S1
Legends for movies S1 and S2

Other Supplementary Material for this manuscript includes the following:

Movies S1 and S2

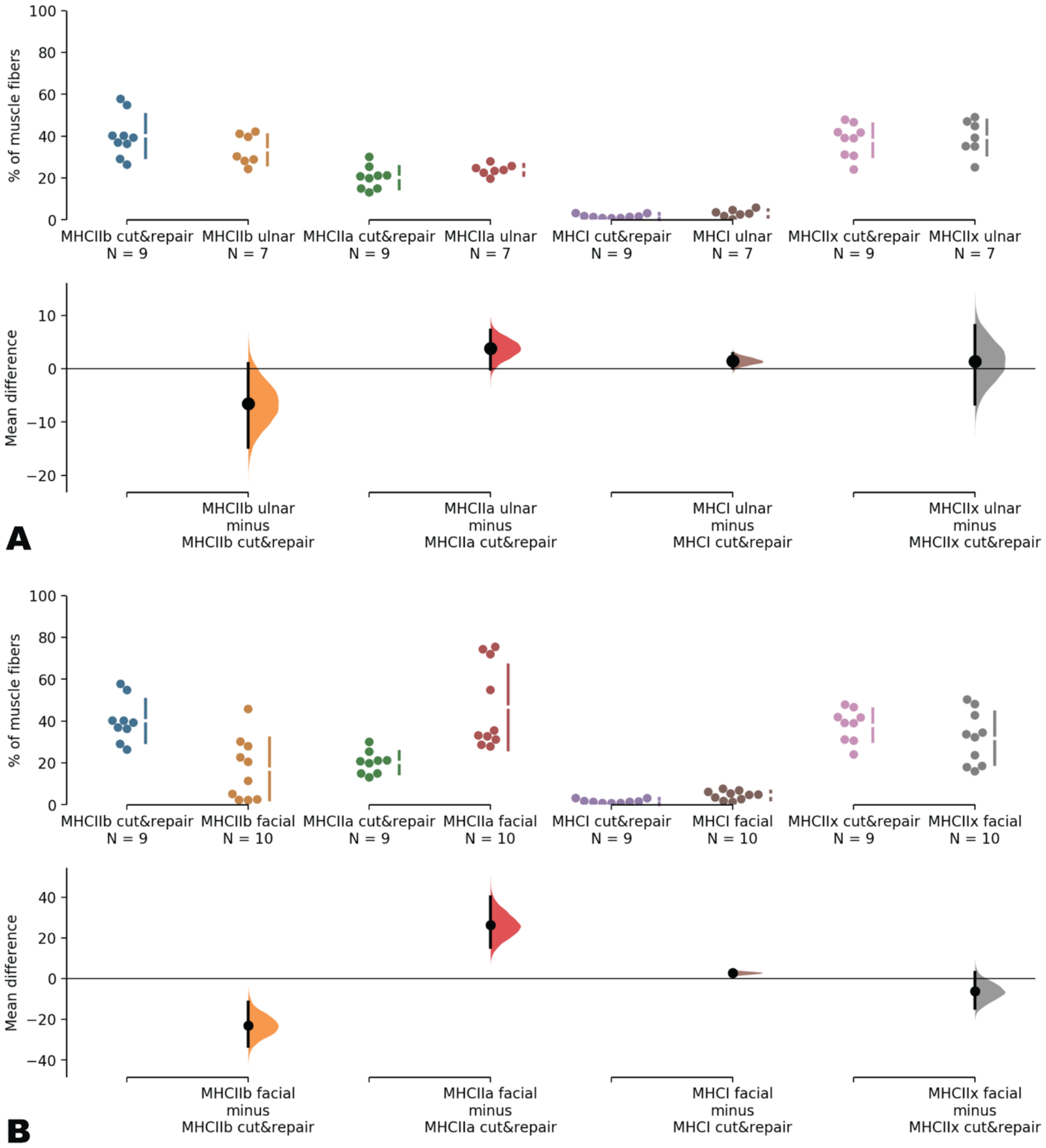

Supplementary Figure 1. Cumming estimation plots for ratio of different muscle fiber types. The individual values are plotted on the upper axes; each mean difference is plotted on the lower axes as a bootstrap sampling distribution. Mean differences are depicted as dots; 95% confidence intervals are indicated by the ends of the vertical error bars**. A.** Comparison between cut&repair and ulnar nerve group. No significance change of mean difference was observed in any of the muscle fiber types: MHCIIb (6.59 [95.0%CI -14.8, 1.01], two-sided permutation t-test, p=0.178), MHCIIa (3.79 [95.0%CI -0.0711, 7.18], two-sided permutation t-test, p=0.11), MHCI (1.44 [95.0%CI 0.0578, 2.84], two-sided permutation t-test, p=0.0642), MHCIIx (1.36 [95.0%CI -6.66, 8.15, two-sided permutation t-test, p=0.735]. **B.** Comparison between cut&repair and facial nerve group. Increase of MHCIIa (26.4 [95.0%CI 15.3, 40.2], two-sided permutation t-test,

p=0.0004) and MHCI (2.85 [95.0%CI 1.41, 4.15], two-sided permutation t-test, p=0.004) as well decrease of MHIIb (-23.0 [95.0%CI -33.2, -11.4, two-sided permutation t-test, p=0.002]. was significant in the facial nerve group compared to the cut&repair group.

Movie S1: Electrical stimulation of the mandibular nerve transferred to the sternomastoid muscle. Muscle contractions are elicited under different twitch frequencies [single twitch, 10, 20, 30, 40, 50 Hz]. The sequence of the muscle contractions corresponds with the sequence of the twitch frequencies as indicated above.

Movie S2: Electrical stimulation of the ulnar nerve transferred to the sternomastoid muscle. Muscle contractions are elicited under different twitch frequencies [single twitch, 10, 20, 30, 40, 50 Hz]. The sequence of the muscle contractions corresponds with the sequence of the twitch frequencies as indicated above.
